# Association Between Preoperative Factors and In-hospital Mortality in Neonates After Cardiac Surgery in China

**DOI:** 10.3389/fped.2021.670197

**Published:** 2021-08-05

**Authors:** Renjie Hu, Hongbin Zhu, Lisheng Qiu, Haifa Hong, Zhiwei Xu, Haibo Zhang, Hao Chen

**Affiliations:** Department of Cardiothoracic Surgery, Shanghai Children's Medical Center, School of Medicine, Shanghai Jiao Tong University, Shanghai, China

**Keywords:** neonate, cardiac surgery, in-hospital mortality, risk factor, low- and middle-income countries

## Abstract

**Background:** Little is known about preoperative factors affecting cardiac surgery outcomes of neonates in China. We sought to examine the association between characteristics of neonates with congenital heart disease (CHD) and early postoperative outcomes after cardiac repair in a tertiary care paediatric hospital.

**Methods:** A single-centre retrospective cohort study of neonates who underwent cardiac surgery between January 2006 and December 2019 was performed. Demographic, institutional, and surgical characteristics of neonates were examined and their association with in-hospital mortality was analysed using multivariable logistic regression models.

**Results:** During the study period, we analysed the outcomes of 1,078 neonates. In-hospital mortality decreased to 13.8% in the era 2017–2019. The overall in-hospital mortality rate was 16.3%. Normal weight at surgery [odds ratio (OR), 0.63; 95% confidence interval (CI), 0.47–0.85; *P* = 0.003] was associated with lower mortality risk. Poor health status (emergent: OR, 3.11; 95% CI, 1.96–4.94; *P* < 0.001; elective: OR, 1.63; 95% CI, 1.11–2.40; *P* = 0.013), higher Society of Thoracic Surgeons-European Association for Cardio-Thoracic Surgery (STAT) categories (STAT 5 category: OR, 2.58; 95% CI, 1.04–6.43; *P* = 0.042), and limited individual surgeon experience (surgeon with 5–10 operations per year: OR, 1.43; 95% CI, 1.06–1.95; *P* = 0.021) were associated with higher odds of early death.

**Conclusion:** In-hospital mortality after neonatal cardiac surgery remained high in our centre over the past 10 years. Some preoperative aspects, including low-weight at surgery, poor health status, increased surgical complexity, and limited surgeon experience were significantly associated with higher mortality. Based on the observed associations, the necessary practises to be modified, especially in preoperative care, should be identified and assessed in future research.

## Introduction

The prevalence of congenital heart disease (CHD) in neonates remains a significant public health threat that requires intensive surveillance. Although surgical care for CHD is rapidly evolving and early mortality has dramatically decreased over time in low- and middle-income countries (LMICs), the management of neonates with CHD remains challenging (1–3). A multi-centre study of the International Quality Improvement Collaborative for Congenital Heart Surgery demonstrated that early mortality of neonates with transposition of the great arteries (TGA) in LMICs was five times higher than that in high-income countries (HICs) (4). Recently, a comprehensive report based on the Global Burden of Diseases, Injuries, and Risk Factors Study showed that the mortality of neonates with severe forms of CHD in LMICs was more than double that of HICs (5).

Risk factors affecting neonatal mortality after cardiac surgery are multifactorial. Several variables, including low weight at surgery, small for gestational age, presence of extracardiac malformations, surgical complexity, and hospital volume, have been associated with higher mortality (6–9). However, most of these data are from centres in HICs, and the risk factors in that specific population remain controversial. Notably, wide variations in medical practises between LMICs and HICs might suggest inherent discrepancies in outcomes. The possibility that there may be unique determinants of increased mortality in LMICs has not been adequately investigated.

Identification of specific factors in resource-constrained LMIC environments may be beneficial for addressing existing deficiencies. As an important limitation, preoperative care in LMICs lags behind that in HICs (10). To our knowledge, little is known about cardiac surgery outcomes for neonates in China, a representative middle-income country, particularly regarding the potential role played by preoperative care. The purpose of this study was to identify the relationship between preoperative characteristics and postoperative in-hospital outcomes among neonates who underwent cardiac surgery in a Chinese institution. Moreover, trends in CHD prevalence and postoperative in-hospital mortality were evaluated to highlight the changes in surgical outcomes over the study period.

## Methods

### Study Population

Neonates (≤ 28 days of age at admission) undergoing cardiothoracic surgery between January 2006 and November 2019 at the Department of Cardiothoracic Surgery, Shanghai Children's Medical Center, were prospectively enrolled. Neonates who underwent pulmonary cystectomy, mediastinal tumour resection, permanent pacemaker implantation, ductus arteriosus closure alone, and those with missing data were excluded. Cases receiving preoperative extracorporeal membrane oxygenation or balloon atrial septostomy were exceedingly rare and were also excluded. The Institutional Review Board at Shanghai Children's Medical Center approved this study. The requirement of informed patient consent was waived because no intervention and further examination was performed.

### Data Collection

Perioperative data were retrospectively collected by reviewing the hospital records and computerised database of our department. Variables for analysis included demographics, diagnoses, preoperative health status, and surgical procedures. Each patient was assigned a primary diagnosis based on the severity of anatomical features. Further subgroupings were used for lesions sharing common pathophysiological characteristics (e.g., left-to-right shunt) whenever possible. Patient-level variates included sex, age, weight at surgery, and history of prematurity. For descriptive purposes, the ages of the subjects were subdivided into 0–7 and 8–28 days. Weight at surgery was subdivided into low-weight (≤ 2.5 kg) and normal-weight (>2.5 kg). Data on chromosomal anomalies were rarely recorded in the database and thus, were not included in our primary analyses.

Preoperative information about hospital factors, including mechanical ventilation, inotrope infusions, and health status, was collected for each patient. Health status was classified as emergent, urgent, or elective (9). Emergent patients were those who underwent surgery within 48 h of admission for a declining medical status. Urgent patients were defined as patients who were admitted in a stable situation and received timely surgical intervention in the same time. In contrast, elective patients were defined as patients who were scheduled for their surgical procedure on an elective basis 48 h later during hospitalisation.

Data regarding the surgical procedures were also reviewed. The surgical risk was calculated according to the Society of Thoracic Surgeons-European Association for Cardio-Thoracic Surgery (STAT) risk categories (6). Referring surgeons were categorised into three groups based on the total number of cardiac operations they have performed per year during the 14-year study period: <5 cases per year, 5–10 cases per year, and >10 cases per year (4).

### Outcome and Assessments

In-hospital death was defined as death during the hospital stay or within 30 days after surgery if the patient was discharged from the hospital. Furthermore, postoperative complications within 30 days after surgery, including infection, dialysis, and unplanned reintervention events were recorded. Unplanned reintervention events were defined as unscheduled catheter-based or surgical treatments following the first surgery for persistent hypoxemia, complete atrioventricular block, secondary bleeding, wound infection or dehiscence, diaphragmatic paralysis, and/or mechanical circulatory support. To compare mortality rates throughout the study period, the surgical era was classified into three timeframes: early (2006–2012), middle (2013–2016), or late (2017–2019). The primary analyses included all neonates who underwent cardiac surgery. Sub-analyses were further performed to evaluate the in-hospital mortality in patients who underwent both cardiopulmonary bypass and aortic clamp procedure.

### Statistical Analysis

Continuous variables were expressed as the median and interquartile range (IQR) according to their distribution. The Wilcoxon test was used to analyse skewed variables. Categorical variables were expressed as frequencies and percentages unless otherwise noted. The chi-square test or Fisher's exact test was used to determine if significant differences existed between groups. The proportion of cases with adverse outcomes was reported, along with a 95% confidence interval (CI).

Possible risk factors were initially analysed using univariate logistic regression. Variables with a *P*-value < 0.1 were introduced into the multivariable logistic regression model. Multivariate analysis was performed to evaluate the effect of preoperative factors in patients undergoing both cardiopulmonary bypass and aortic clamp procedure. A forest plot of in-hospital mortality was created to describe the results of multivariable logistic regression. *P*-values and 95% CIs were reported for the overall comparison and in the subgroups of interest. Odds ratios (ORs) and 95% CIs were calculated to evaluate the strength of any association. Statistical significance was set at ≤ 0.05 for two-sided *P*-values. The results were analysed using commercially available statistical software (SPSS 24, IBM, Armonk, NY, USA; Stata 15, StataCorp, College Station, TX, USA).

## Results

### Patient Characteristics

**Figure 1:**
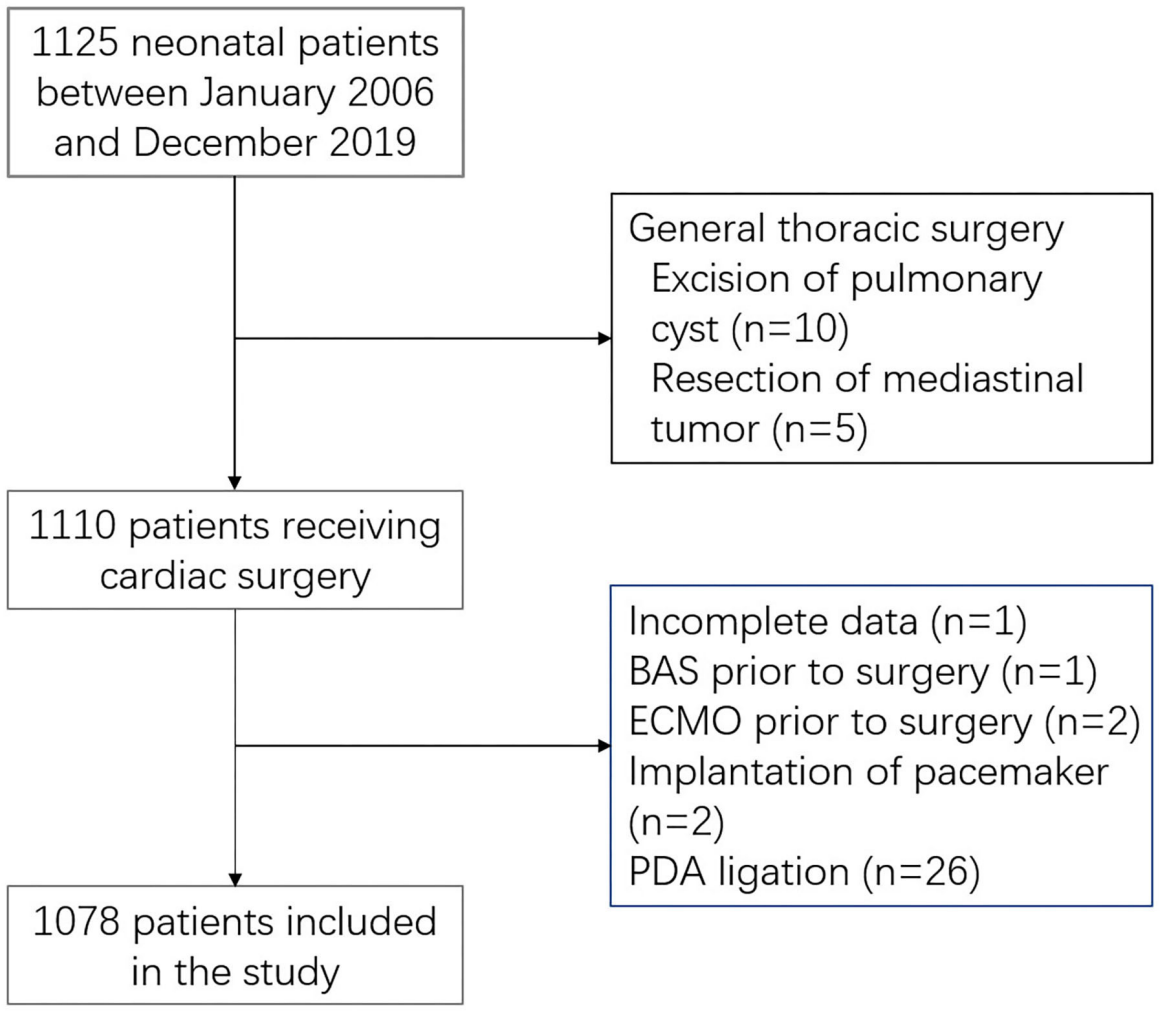
Study population. BAS, balloon atrial septostomy; PDA, patent ductus arteriosus; ECMO, extracorporeal membrane oxygenation.

Over the study period, 1,125 neonates were assessed, and 1,078 (95.8%) met the inclusion criteria (Figure 1). The patient characteristics are summarised in Table 1. Of 1,078 patients, 73.5% were male and 8.1% were born prematurely. The median age at surgery was 13 days (IQR, 7–20 days). The median weight was 3.4 kg (IQR, 3.0–3.7 kg), with 8.6% having low body-weight. The detailed distribution of diagnoses is shown in Supplementary Table 1.

**Table 1:** Preoperative characteristics for 1,078 study patients.

	**In-hospital death (*n* = 176)**	**Discharged alive (*n* = 902)**	***P*-value**
**PATIENT FACTORS**			
Male sex, *n* (%)	119 (67.6)	673 (74.6)	0.054
Prematurity, *n* (%)	22 (12.5)	65 (7.2)	0.018
Age, median (IQR)	11 (6–18)	13 (7–21)	0.016
0–7 d, *n* (%)	60 (34.1)	244 (27.1)	0.058
Weight, median (IQR)	3.2 (2.8-3.5)	3.4 (3.0-3.7)	<0.001
≤ 2.5 kg, *n* (%)	22 (12.5)	71 (7.9)	0.045
**Primary diagnosis**, ***n*****(%)**
Conotruncal defects	81 (46.0)	449 (49.8)	0.362
Left heart lesions	44 (25.0)	275 (30.5)	0.145
Right heart lesions	34 (19.3)	135 (15.0)	0.146
Univentricular heart lesions	13 (7.4)	23 (2.5)	0.001
Left to right shunt	4 (2.3)	20 (2.2)	1.000
**HOSPITAL FACTORS**			
Inotropic agents, *n* (%)	57 (32.4)	177 (19.6)	<0.001
Mechanical ventilation, *n* (%)	79 (44.9)	267 (29.6)	<0.001
**Health status**, ***n*****(%)**
Emergent	64 (36.4)	134 (14.9)	<0.001
Urgent	35 (19.9)	364 (40.4)	<0.001
Elective	77 (43.8)	404 (44.8)	0.800
**SURGICAL FACTORS**			
**STAT category**, ***n*****(%)**
1	1 (0.6)	14 (1.6)	0.488
2	14 (8.0)	93 (10.3)	0.339
3	56 (31.8)	349 (38.7)	0.085
4	97 (55.1)	440 (48.8)	0.124
5	8 (4.5)	6 (0.7)	0.001
**Surgeon experience**, ***n*****(%)**
<5 cases per year	8 (4.5)	70 (7.8)	0.132
5–10 cases per year	123 (69.9)	527 (58.4)	0.004
>10 cases per year	45 (25.6)	305 (33.8)	0.033

*IQR, interquartile range; STAT, Society of Thoracic Surgeons-European Association for Cardio-Thoracic Surgery*.

In terms of preoperative care, 32.1% of patients underwent mechanical ventilation and 21.7% were administered inotropes. The median time from admission to surgery was 3 days (IQR, 1–6 days), with 18.4% of neonates admitted in emergency status, 37% in urgent status, and 44.6% in elective status. All surgical procedures were classified according to STAT risk categories, and most were Category 4 (49.8%), followed by Category 3 (37.6%), and Category 2 (9.9%). The median number of neonatal surgical operations performed by each surgeon annually was 27 (IQR, 2–95), yet most surgeons (67.5%) performed fewer than 10 cases annually.

**Figure 2:**
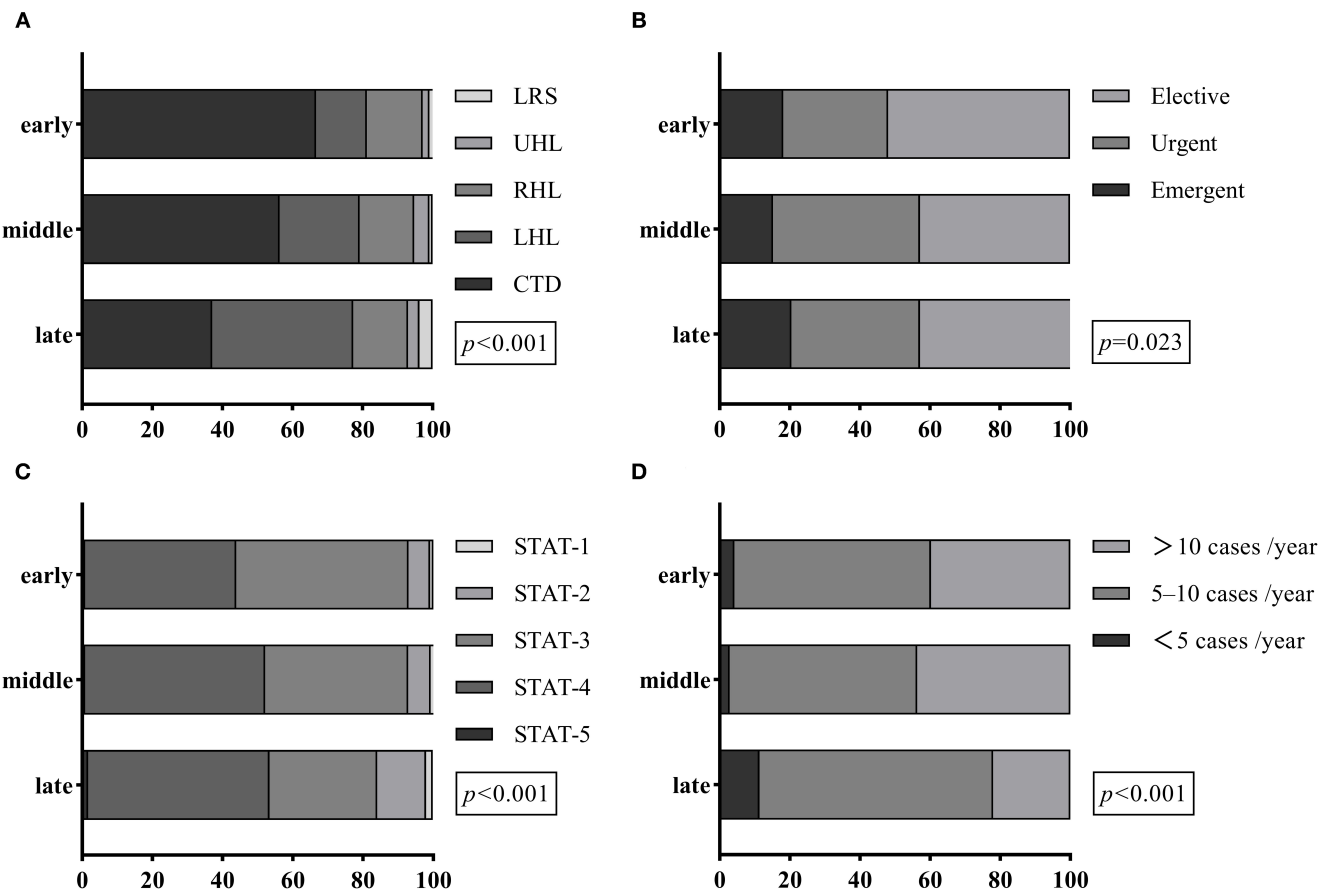
Trend analysis for the prevalence of primary diagnosis **(A)**, health status **(B)**, STAT category **(C)**, and surgeon experience **(D)** in different surgical eras. CTD, conotruncal defects; LHL, left heart lesions; LRS, left to right shunt; RHL, right heart lesions; STAT, Society of Thoracic Surgeons-European Association for Cardio-Thoracic Surgery; UHL, univentricular heart lesions.

The number of neonates and the total number of children operated during the study period are shown in Supplementary Figure 1. In the late timeframe (2017–2019), major changes were observed in the constitution of CHD (*P* < 0.001), STAT categories (*P* < 0.001), health status (*P* = 0.023), and experience of surgeons (*P* < 0.001) (Figure 2). Left heart lesions peaked at 40.3% of the cohort in the late era. However, the proportion of conotruncal defects dropped to 37.1% in the late era, which is two-thirds of defects in the early. Furthermore, there was a trend toward a shorter time of admission (emergent or urgent status) and more complex operations (STAT Categories 4 and 5). Impressively, the overall surgical volume performed by surgeons with less experience (annual surgical volume less than 10 cases) accounted for almost 80% in the most recent era.

### Clinical Outcomes

Postoperatively, 53.8% (IQR, 50.8–56.8%) of patients required delayed sternal closure, and 19.4% (17.0–21.8%) required dialysis. Among patients who stayed in the intensive care unit, 10.7% (8.8–12.5%) developed a nosocomial infection and 12.9% (10.9–14.9%) underwent unplanned reintervention. In-hospital mortality was 16.3% (95% CI, 14.1–18.5%). The median hospital length of stay was 17 days (IQR, 13–23 days), and postoperative length of stay was 13 days (IQR, 10–17 days).

**Figure 3:**
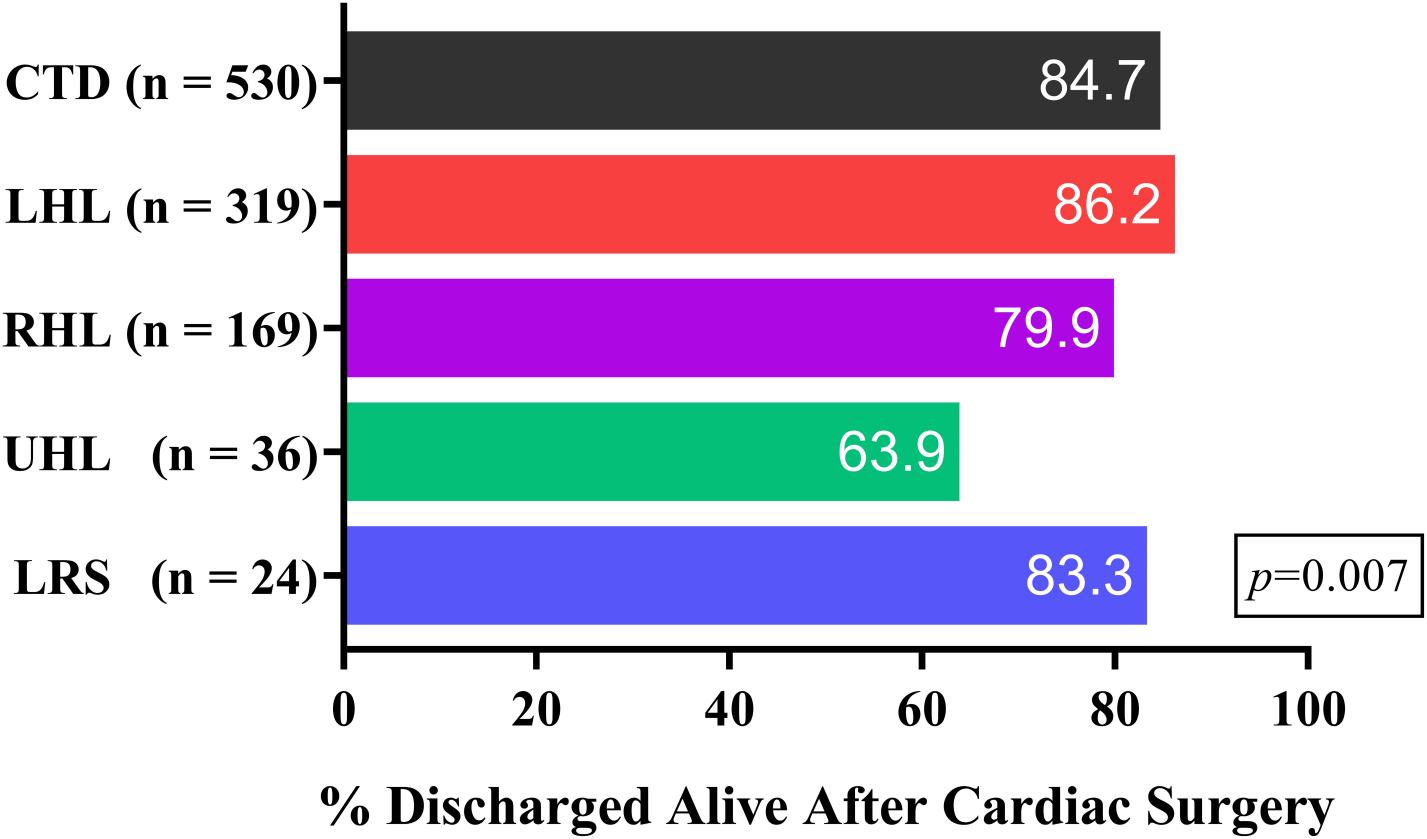
Early survival rates in different group of primary diagnosis. CTD, conotruncal defects; LHL, left heart lesions; LRS, left to right shunt; RHL, right heart lesions; UHL, univentricular heart lesions.

In-hospital mortality rates stratified by primary diagnosis, health status, STAT category, and surgeon experience are presented in Table 2. Early survival rates in different group of primary diagnosis are illustrated in Figure 3. Mortality was significantly increased in the more complicated CHD groups (*P* = 0.007) and higher STAT categories (*P* < 0.001). Mortality in the univentricular pathway was 36.1 and 57.1% in Category 5. Poor health status (*P* < 0.001) and limited surgeon experience (*P*=0.015) were also significantly associated with mortality. Mortality was 8.8%, 16.0 and 32.3% in patients in urgent, elective and emergent status, respectively. Strikingly, mortality in the surgeon group of 5–10 cases per year was higher (18.9%) than that in the groups with <5 and >10 cases per year.

**Table 2:** In-hospital mortality of cardiac surgery in neonates stratified by primary diagnosis, health status, STAT category, and surgeon experience.

**Primary diagnosis**	**CTD**	**LHL**	**RHL**	**UHL**	**LRS**	***P*-value**
Early mortality	81/530 (15.3)	44/319 (13.8)	34/169 (20.1)	13/36 (36.1)	4/24 (16.7)	0.007
**Health status**	**Emergent**		**Urgent**	**Elective**		***P*** **-value**
Early mortality	64/198 (32.3)		35/399 (8.8)	77/481 (16.0)		<0.001
**STAT category**	**1**	**2**	**3**	**4**	**5**	***P*** **-value**
Early mortality	1/15 (6.7)	14/107 (13.1)	56/405 (13.8)	97/537 (12.8)	2/24 (18.1)	<0.001
**Surgeon experience**	** <5 cases per year**		**5–10 cases per year**	**>10 cases per year**		***P*** **-value**
Early mortality	8/78 (10.3)		123/650 (18.9)	45/350 (12.9)		0.015

*CTD, conotruncal defects; LHL, left heart lesions; LRS, left to right shunt; RHL, right heart lesions; STAT, Society of Thoracic Surgeons-European Association for Cardio-Thoracic Surgery; UHL, univentricular heart lesions*.

When examining the era effect, there was a decrease in-hospital mortality (*P* = 0.297, Supplementary Figure 2) through time. The increasing survival rates after discharge were noted for most cardiac lesions and almost all STAT categories in the more recent era, although the difference was not statistically significant (all *P* ≥ 0.05). The mortality rate in conotruncal defects, including TGA with an intact ventricular septum, TGA with ventricular septal defect and Taussig-Bing anomaly, was observed to be a notable exception (early, 14.0%; middle, 15.4%; and late, 16.1%).

### Associations Between Preoperative Characteristics and Outcomes

**Figure 4:**
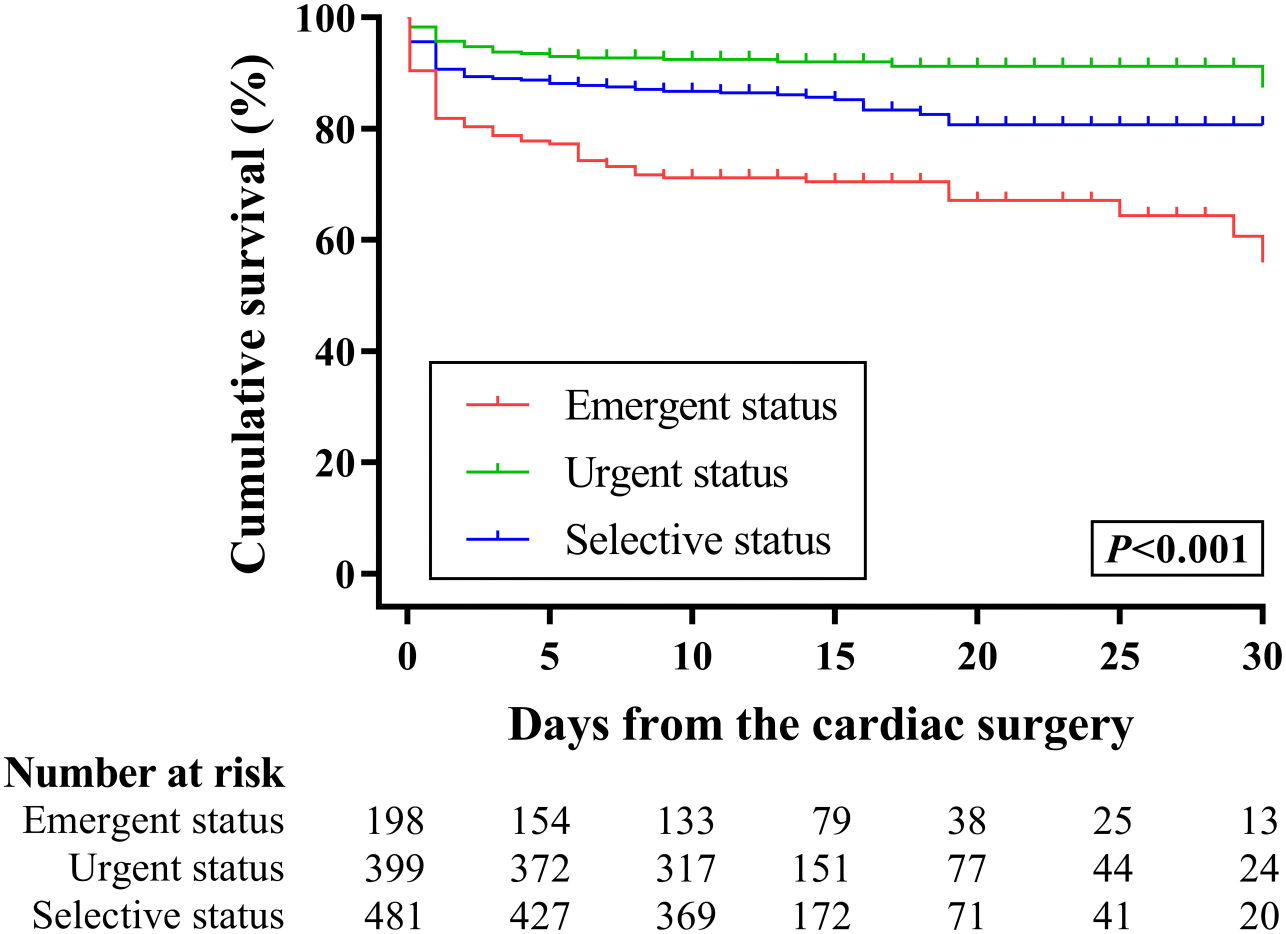
Kaplan-Meier analysis for the 30-day survival of neonates with congenital heart diseases according to their preoperative health status.

A multivariable model evaluated preoperative factors associated with in-hospital mortality (Table 3). Preoperative emergent status (OR, 3.11; 95% CI, 1.96–4.94; *P* < 0.001), elective status (OR, 1.63; 95% CI, 1.11–2.40; *P* = 0.013), STAT Category 5 (OR, 2.58; 95% CI, 1.04–6.43; *P* = 0.042), and surgeons with 5–10 cases per year (OR, 1.43; 95% CI, 1.06–1.95; *P* = 0.021) were associated with higher odds of death, whereas increased weight at surgery was associated with lower odds of mortality (OR, 0.63; 95% CI, 0.47–0.85; *P* = 0.003). Preoperative administration with inotropes (*P* = 0.15) and mechanical ventilation (*P* = 0.52) were not associated with mortality, as well as age at surgery (*P* = 0.147) and premature delivery (*P* = 0.509). Figure 4 shows the Kaplan-Meier survival curves for patients with different preoperative health statuses. Conditional logistic regression showed that urgent status was associated with lower 30-day mortality, compared with emergent, and elective status (*P* < 0.001).

**Table 3:** Multivariable model of preoperative factors associated with in-hospital mortality.

	**Mortality**	
	**OR (95% CI)**	***P* value**
Age	0.97 (0.97–1.00)	0.147
Weight	0.63 (0.47–0.85)	0.003
Female, sex	1.23 (0.92–1.64)	0.156
Prematurity	0.82 (0.44–1.50)	0.509
Inotropic agents	1.26 (0.92–1.74)	0.150
Mechanical ventilation	0.88 (0.60–1.30)	0.520
**Health status**		
Urgent	Reference	
Emergent	3.11 (1.96–4.94)	<0.001
Elective	1.63 (1.11–2.40)	0.013
**STAT category**		
2	Reference	
5	2.58 (1.04–6.43)	0.042
**Surgeon experience**		
>10 cases per year	reference	
5–10 cases per year	1.43 (1.06–1.95)	0.021

*CI, confidence interval; OR, odds ratio; STAT, Society of Thoracic Surgeons-European Association for Cardio-Thoracic Surgery*.

**Figure 5:**
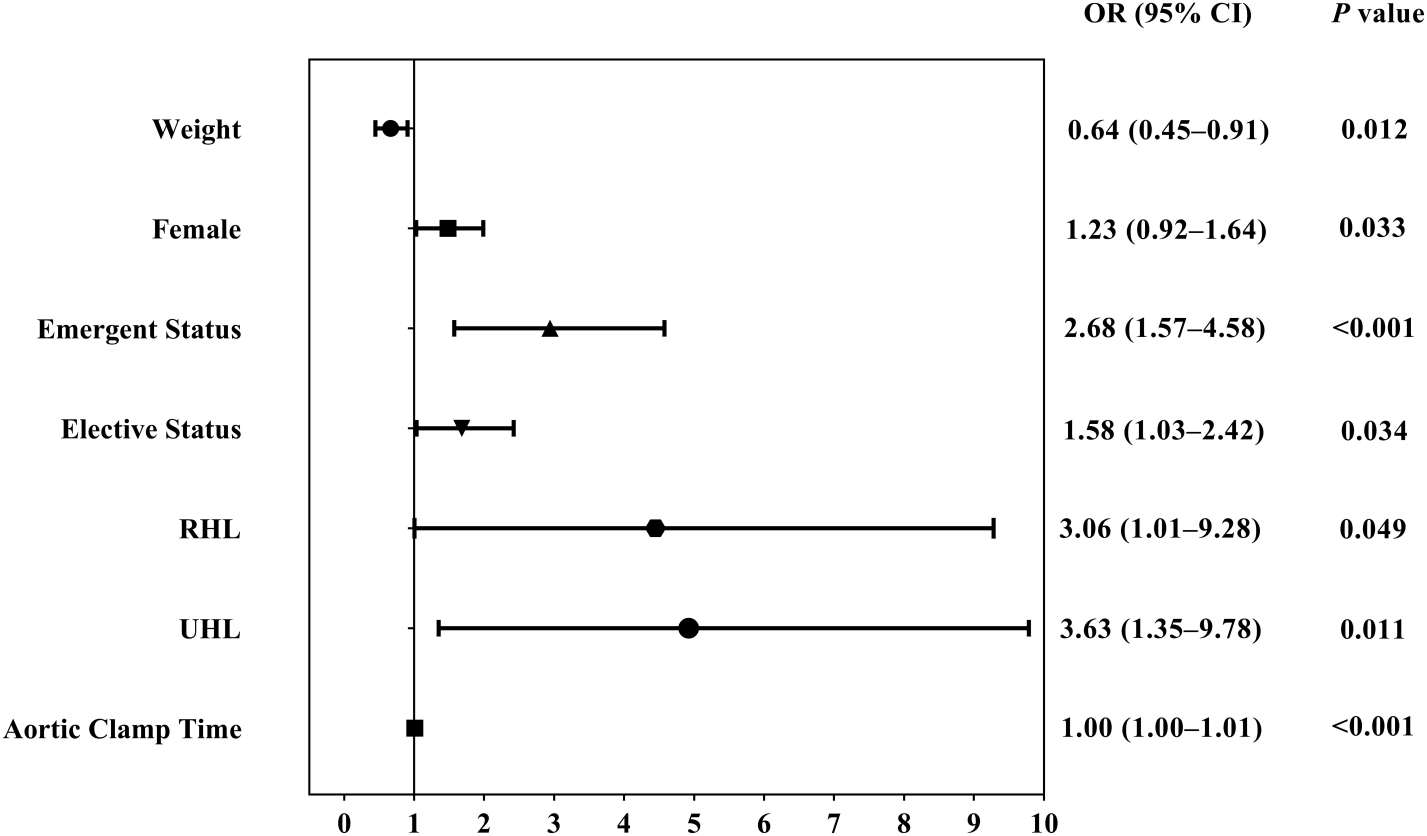
Forest plot showing adjusted OR of factors associated with in-hospital mortality in patients experiencing both cardiopulmonary bypass and aortic clamp procedure. CI, confidence interval; OR, odds ratio, RHL, right heart lesions; UHL, univentricular heart lesions.

After exclusion of the patients who did not undergo the aortic clamp procedure, the multivariate analysis showed that factors associated with increased in-hospital mortality were female sex (OR, 1.23; 95% CI, 0.92–1.64; *P* = 0.033), emergent status (OR, 2.68; 95% CI, 1.57–4.58; *P* < 0.001), elective status (OR, 1.58; 95% CI, 1.03–2.42; *P* = 0.034), right heart lesions (OR, 3.06; 95% CI, 1.01–9.28; *P* = 0.049), univentricular heart lesions (OR, 3.63; 95% CI, 1.35–9.78; *P* = 0.011), and aortic clamp time (OR, 1.0; 95% CI, 1.0–1.01; *P* < 0.001) (Supplementary Table 2). In-hospital mortality remained significantly higher in patients who underwent cardiac surgery in emergent (29.6%) or elective (15.6%) status than that in urgent status (8.4%; *P* < 0.001). Figure 5 shows the forest plot for the adjusted OR of each factor included in the multivariate analysis.

## Discussion

This study reports the clinical outcomes of neonates who underwent cardiac surgeries in Shanghai Children's Medical Center, a Chinese paediatric hospital in a resource-constrained middle-income country, as observed over 14 years. This study evaluated the preoperative factors associated with in-hospital mortality. We found an improvement in postoperative in-hospital mortality rates after neonatal cardiac surgery in our department. Health status, as an important variable of perioperative care, was independently associated with in-hospital mortality, especially in neonates undergoing cardiopulmonary bypass and aortic clamp procedure.

Despite major progress in surgical techniques and monitoring equipment during the past two decades, the in-hospital mortality of our department was still significantly higher than that of most contemporary HICs (7, 11–14). The mortality rate of 16.3% in this study is an important reminder that some conditions in resource-constrained LMIC environments might carry inherent risks. Factors affecting outcomes are likely heterogeneous and vary at the geographic, socioeconomic, hospital, and patient levels. It is crucial to recognise that there is still much room for improvement in LMICs.

Preoperative health status, a comprehensive reflection of the quality of neonatal management, is one of the most important factors affecting surgical outcomes. In the present study, 18.4% of patients who underwent emergent surgeries within 48 h after admission for unstable conditions had a significantly increased mortality, which underlined the importance of good preoperative status. The factors affecting poor and deteriorating health status in neonates in LMICs are complex. Lack of prenatal diagnosis, inadequate number of paediatric cardiologists in community hospitals, and undeveloped transfer systems may be responsible in the pre-hospital setting (15–17). More importantly, there is great uncertainty about the optimal preoperative in-hospital care for neonates awaiting cardiac surgery. There remains a lack of accurate risk stratification models that may improve surgical outcomes through the identification of potential outcome determinants in an LMIC setting.

In our study, the use of prostaglandin infusion, inotropes, and mechanical ventilation before surgery was performed consistent with practises in HICs. However, more aggressive interventions to provide physiologically favourable attributes to atrial septal defects and patent ductus arteriosus cannot be accessed easily. Consequently, a preceding septostomy was rarely performed in our centre. Patients who underwent this intervention were excluded from this study. Similarly, this practise was found to be only used in a comparatively small group of cases in other LMIC healthcare institutions (4, 18). Conversely, a septostomy is considered a valuable aspect of preoperative care in HICs and is performed frequently (4, 19). This difference not only suggests the lack of adequate catheterisation equipment, facilities, and expertise but also reflects a significant cognitive bias in LMICs. For instance, most paediatric cardiac surgeons in China prefer to undertake a primary repair than to perform a relatively safe staged operation for economic reasons.

To date, the optimal timing of cardiac surgery in neonates presenting with stable preoperative status remains controversial. A recent review showed that the majority of neonates with CHD can be operated on early with better results compared to medical management or palliation (20). It has also been documented that in neonates with TGA, earlier arterial switch operation was not associated with a higher risk of early mortality (19, 21, 22). However, some unfavourable preoperative characteristics, including low birth weight, prematurity, and being small for gestational age, could delay the timing of surgery. Conventional management of these patients continues to be the deferral of corrective surgery using aggressive medical management or palliative surgery until certain stability is achieved. Unfortunately, the optimal duration of delay has not been clearly defined. This approach of delaying surgery could bring tremendous uncertainty for these neonates and eventually increase the risk for infections, gastrointestinal ischemia, and worsening hemodynamic status. Unsurprisingly, patients with elective status in our study were found to be associated with higher in-hospital mortality compared to those with urgent status.

Compared to low weight or small for gestational age, the severity of lesions, and the complexity of surgical procedures were more widely accepted as significant predictors of early mortality after cardiac surgery in neonates. We found that clinical outcomes in this report were similar to those identified by the Society of Thoracic Surgeons in that the in-hospital mortality increased with higher STAT categories and Category 5 was associated with the highest risk (11–14, 23). Additionally, a longer aortic cross-clamp time was considered an important risk factor for in-hospital mortality. An increased aortic cross-clamp time was found to be consistent with a more complex cardiovascular malformation, leading to a technically demanding repair and subsequently, prolonged circulatory support. Another notable similarity between our centre and most settings in the United States and Europe is that increasing surgeon or hospital experience is associated with better clinical outcomes (24, 25). In the present study, surgeons with annual neonatal surgical volumes exceeding 10 cases displayed a lower risk of operative mortality. The significant variation in morbidities incurred among surgeons undoubtedly reflects a discrepancy in the learning curve for the operative care of neonates.

Care must be taken in interpreting observed trends in CHD during the past 10 years in our department. First, the remarkable growth of left heart lesions, represented by total anomalous pulmonary venous drainage, has dramatically changed the constitution of CHDs and raised the complexity of preoperative diagnosis. Second, more complex surgical procedures (STAT Categories 4 and 5) have emerged and have contributed to an increase in operative difficulty. Third, although all surgeons who participated in this study had considerable operative experience, operations performed by more experienced surgeons accounted for only one-third of all surgeries. Furthermore, the number of surgeons included in this study greatly exceeded that in studies from centres in HICs. However, one database from HICs was only linked to a hospital cardiac surgery program, and uncommonly, to an individual cardiac surgeon (19, 24, 25). Taken together, with an increasing number of neonates undergoing cardiac surgery, more strategic modifications should be considered, and greater efforts should be made in the current and future era.

This study has limitations. First, it is a non-randomised retrospective study that has a limited statistical power. Second, restricted by the scope of our database, details regarding the prenatal diagnosis, presence of genetic syndrome, and importantly, nutritional and infective history were not captured completely. Therefore, the interplay between these factors and early surgical outcomes cannot be comprehensive. Our models were adjusted for only the factors that are captured in the database. Finally, this was a single-centre study, and there may be some selection bias that could confound the results. Thus, we could not provide much insight into the therapeutic strategies that might be advantageous for given clinical situations. The following are important factors to note for the study, in terms of possible confounders: (1) Our cohort extended across a period that included substantial advances in surgical technology and intensive care treatment; (2) The sex ratio of the study population was severely unbalanced, which meant that more male patients were preferentially referred to the hospital; (3) Patients rarely underwent a preceding catheter treatment before surgical repair in our centre, and some patients lacking physiologically favourable attributes had to be put in aggravated preoperative health status; and (4) Although most experienced surgeons undertook most complex cases in this study, majority of operations were performed by surgeons with 5–10 operations per year. This situation may lead to in-hospital mortality in some types of CHD remains in a high level.

Although in-hospital mortality of neonatal cardiac surgery has improved in the past 14 years in our centre, it is still notably higher than that in HICs. Weight at surgery, preoperative health status, STAT category, and surgeon experience were associated with mortality. Compared to emergent or elective status, preoperative urgent status was associated with a significantly decreased risk of mortality. Trends in the prevalence and mortality of patients were also evaluated. These findings suggest that greater effort should be made in the management of preoperative care to improve outcomes of neonates in LMIC environments.

## Data Availability Statement

The original contributions presented in the study are included in the article/Supplementary Material, further inquiries can be directed to the corresponding author/s.

## Ethics Statement

The studies involving human participants were reviewed and approved by Institutional Review Board at Shanghai Children's Medical Center, Shanghai Jiao Tong University School of Medicine. Written informed consent from the participants' legal guardian/next of kin was not required to participate in this study in accordance with the national legislation and the institutional requirements.

## Author Contributions

RH conceptualised and designed the study, collected data and drafted the initial manuscript. HZ, LQ, and HH designed the data collection instruments and carried out the initial analyses. HZ and ZX conceptualised and designed the study, and reviewed and revised the manuscript. HC conceptualised and designed the study, coordinated and supervised data collection, and critically reviewed the manuscript for important intellectual content. All authors approved the final manuscript as submitted and agree to be accountable for all aspects of the work.

## Conflict of Interest

The authors declare that the research was conducted in the absence of any commercial or financial relationships that could be construed as a potential conflict of interest.

## Publisher's Note

All claims expressed in this article are solely those of the authors and do not necessarily represent those of their affiliated organizations, or those of the publisher, the editors and the reviewers. Any product that may be evaluated in this article, or claim that may be made by its manufacturer, is not guaranteed or endorsed by the publisher.

## Supplementary Material

Supplementary Figure 1 The number of neonates and the total number of children operated during the study period.

Supplementary Figure 2 Trend of in-hospital mortality in different surgical eras.

Supplementary Table 1 Distribution of primary cardiac defects.

Supplementary Table 2 Multivariable model of preoperative factors associated with in-hospital mortality in neonates undergoing aortic-clamp operation.

## Abbreviations

CI: confidence interval
CHD: congenital heart disease
HICs: high-income countries
IQR: interquartile range
LMICs: low- and middle-income countries
OR: odds ratio
STAT: Society of Thoracic Surgeons-European Association for Cardio-Thoracic Surgery
TGA: transposition of the great arteries.
