# Supplementary figures and images for: Association Between Preoperative Factors and In-hospital Mortality in Neonates After Cardiac Surgery in China
Source: Front Pediatr. 2021 Aug 5;9:670197. doi: 10.3389/fped.2021.670197 (PMC8374182; doi:10.3389/fped.2021.670197)

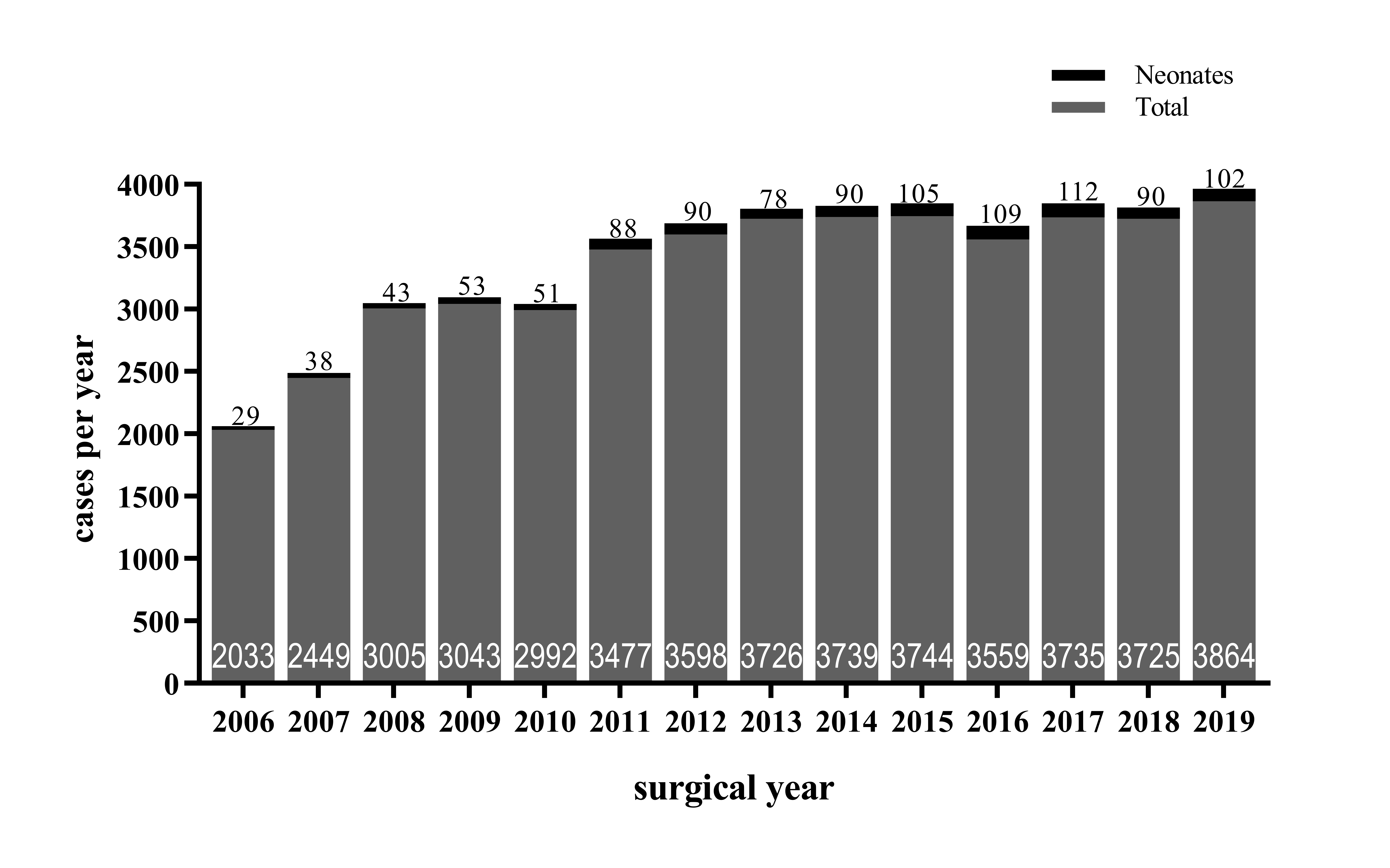

Supplement: Supplementary Figure 1 — The number of neonates and the total number of children operated during the study period. [file Image_1.TIF]

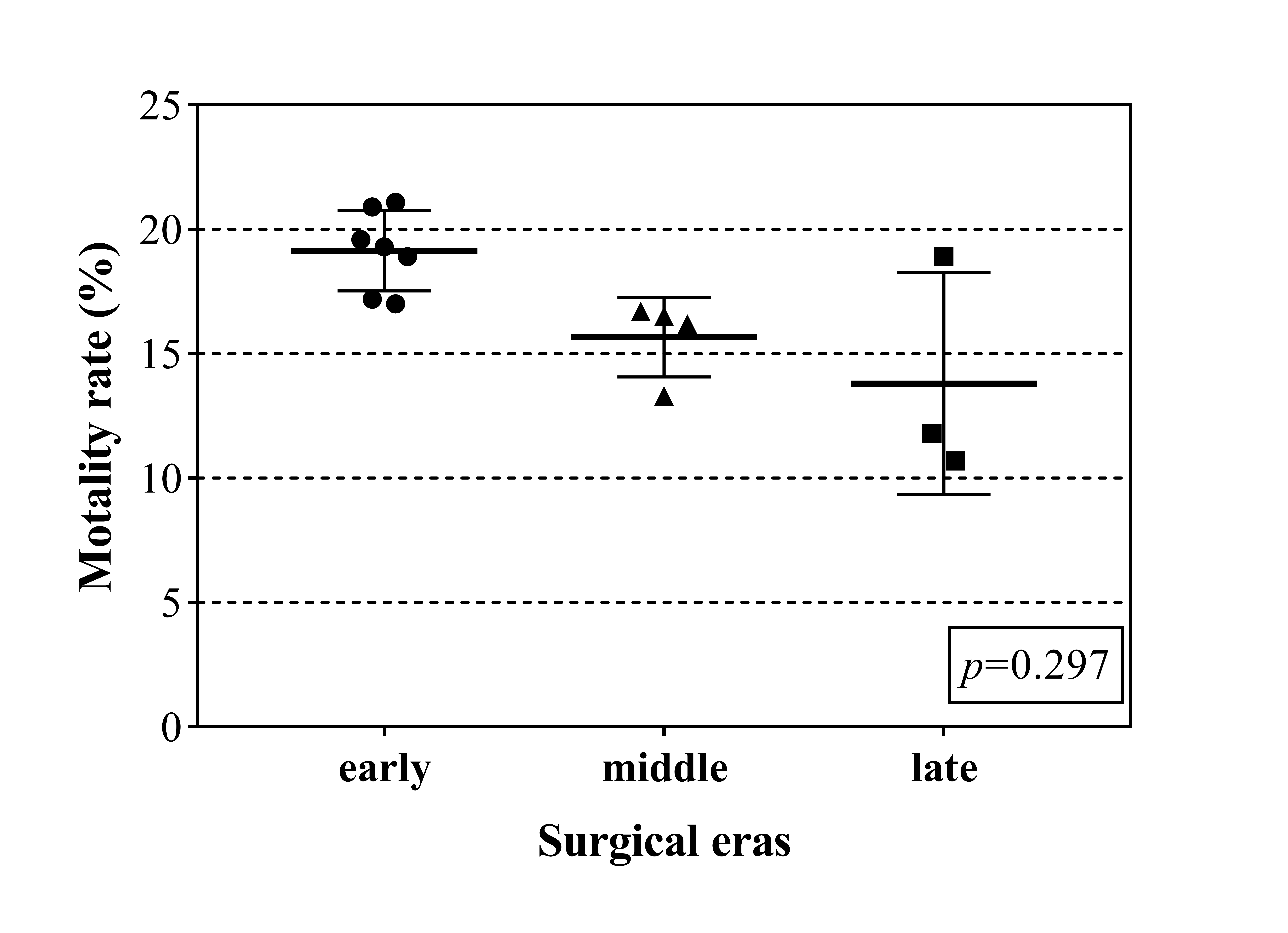

Supplement: Supplementary Figure 2 — Trend of in-hospital mortality in different surgical eras. [file Image_2.TIF]
