# Supplementary material for: Association Between Preoperative Factors and In-hospital Mortality in Neonates After Cardiac Surgery in China
Source: Front Pediatr. 2021 Aug 5;9:670197. doi: 10.3389/fped.2021.670197 (PMC8374182; doi:10.3389/fped.2021.670197)
Supplement: Supplementary Table 1 — Distribution of primary cardiac defects. [file Table_1.DOCX]

**Table S1. Distribution of primary cardiac defects**

|  | **n** | **%** |
| --- | --- | --- |
| Conotruncal defect (CTD) | 530 | 49.2 |
| TGA, IVS | 318 | 29.5 |
| TGA, VSD | 179 | 16.6 |
| TGA, aortic arch hypoplasia | 27 | 2.5 |
| Truncus arteriosus | 6 | 0.6 |
| Left heart lesion (LHL) | 319 | 29.6 |
| Aortic stenosis | 2 | 0.2 |
| Coarctation of aorta | 86 | 8.0 |
| Cor triatriatum | 2 | 0.2 |
| Interrupted aortic arch | 68 | 6.3 |
| Left atrial myxoma | 1 | 0.1 |
| TAPVD | 160 | 14.8 |
| Right heart lesion (RHL) | 169 | 15.7 |
| PA, IVS | 53 | 4.9 |
| PA, VSD | 30 | 3.2 |
| Pulmonary stenosis | 78 | 7.2 |
| Right ventricular tumor | 4 | 0.4 |
| Tetralogy of Fallot | 4 | 0.4 |
| Univentricular heart lesion (UHL) | 36 | 3.3 |
| LV type | 10 | 0.9 |
| RV type | 20 | 1.9 |
| Other | 6 | 0.6 |
| Left to right shunt (LRS) | 24 | 2.2 |
| ASD | 2 | 0.2 |
| Coronary fistula | 1 | 0.1 |
| Hemitruncus | 7 | 0.6 |
| TAVC | 1 | 0.1 |
| VSD | 13 | 1.2 |

ASD, atrial septal defect; IVS, intact ventricular septum; LV, left ventricle; PA, pulmonary atresia; RV, right ventricle; TAPVD, total anomalous pulmonary venous drainage; TAVC, transitional atrioventricular canal; TGA, transposition of the great arteries; VSD, ventricular septal defect.
