# Supplementary material for: Association Between Preoperative Factors and In-hospital Mortality in Neonates After Cardiac Surgery in China
Source: Front Pediatr. 2021 Aug 5;9:670197. doi: 10.3389/fped.2021.670197 (PMC8374182; doi:10.3389/fped.2021.670197)
Supplement: Supplementary Table 2 — Multivariable model of preoperative factors associated with in-hospital mortality in neonates undergoing aortic-clamp operation. [file Table_2.DOCX]

**Table S2. Multivariable model of preoperative factors associated with in-hospital mortality in neonates undergoing aortic-clamp operation**

|  | Mortality | |
| --- | --- | --- |
|  | **OR (95% CI)** | ***P* value** |
| Age | 0.99 (0.97–1.01) | 0.302 |
| Weight | 0.64 (0.45–0.91) | 0.012 |
| Female | 1.23 (0.92–1.64) | 0.033 |
| Prematurity | 1.32 (0.70–2.48) | 0.396 |
| Inotropic agents | 1.20 (0.82–1.75) | 0.356 |
| Mechanical ventilation | 0.90 (0.59–1.39) | 0.635 |
| Health status |  |  |
| Urgent | reference |  |
| Emergent | 2.68 (1.57–4.58) | <0.001 |
| Elective | 1.58 (1.03–2.42) | 0.034 |
| Primary Diagnosis |  |  |
| Conotruncal defects | reference |  |
| Left heart lesions | 1.26 (0.81–1.97) | 0.305 |
| Right heart lesions | 3.06 (1.01–9.28) | 0.049 |
| Univentricular heart lesions | 3.63 (1.35–9.78) | 0.011 |
| Surgeon experience |  |  |
| >10 cases per year | reference |  |
| 5–10 cases per year | 1.26 (0.87–1.80) | 0.218 |
| Aortic Clamp Time | 1.00 (1.00–1.01) | <0.001 |

CI, confidence interval; OR, odds ratio.
